# Hepatitis A and E Virus Seroprevalence and Water, Sanitation and Hygiene Levels in Rural Areas of Khammouane Province, Lao People's Democratic Republic: A Cross‐Sectional Study

**DOI:** 10.1002/jmv.70524

**Published:** 2025-07-30

**Authors:** Vilaysone Khounvisith, Peter Odermatt, Siriphone Virachith, Nouna Innoula, Bounta Vongphachanh, Latdavone Khenkha, Jan Hattendoft, Judith M. Hübschen, Antony P. Black

**Affiliations:** ^1^ LaoLuxLab/Vaccine Preventable Diseases Laboratory Institut Pasteur du Laos Vientiane Lao PDR; ^2^ Swiss Tropical and Public Health Institute Allschwil Switzerland; ^3^ University of Basel Basel Switzerland; ^4^ Department of Infection and Immunity Luxembourg Institute of Health Esch‐sur‐Alzette, Grand‐Duchy of Luxembourg; ^5^ School of life sciences University of Westminster London UK

**Keywords:** hepatitis, Laos, seroprevalence, virus, WASH levels

## Abstract

There have been several Water, Sanitation, and Hygiene (WASH) interventions in Lao People's Democratic Republic (Lao PDR). We aimed to determine the relationship of exposure to two faecal‐orally transmitted pathogens, hepatitis A virus (HAV) and hepatitis E virus (HEV), with WASH levels and other factors. A cross‐sectional study conducted in three districts in Khammouane Province enrolled 2300 participants aged 5 to 87 years by random sampling. Anti‐HAV and anti‐HEV antibodies were detected by enzyme‐linked immunosorbent assay. The relationship between serology (outcome), WASH and other variables was determined by bivariate and multivariable analysis. Overall, 12.0% of participants had surface water as a water source, 22.0% practiced open defecation and 66.9% had basic hygiene facilities. Anti‐HAV IgG seropositivity was 63.2% and 57.5% were anti‐HEV seropositive. The mean age at which 50% of the population were positive for anti‐HAV and anti‐HEV was 24 and 27 years old, respectively. Anti‐HAV seroprevalence was lower in those with improved sanitation than those practicing open defecation (OR = 0.6, 95%CI = 0.4–0.8, *p* = 0.001) and higher in adults consuming undercooked meat (OR = 1.5, 95%CI = 1.1–2.0, *p* = 0.01). It also varied by district, ethnicity, education and age. Anti‐HEV seroprevalence was lower in those with improved water source than those using surface water (OR = 0.6, 95%CI = 0.4–0.8, *p* = 0.002) and higher in adults consuming raw meat (OR = 1.3, 95%CI = 1.0–1.7, *p* = 0.04). Anti‐HEV seroprevalence varied by district, sex, education, and age. Khammouane province has low levels of WASH leading to high transmission of HAV and HEV. Reducing the practice of open defecation and other risk practices such as undercooked meat consumption may reduce transmission as well as consideration of HAV vaccine introduction for younger ages.

**Abbreviations**
CI: confidence intervals
ELISA: enzyme‐linked immunosorbent assay
HAV: hepatitis A virus
HEV: hepatitis E virus
Lao PDR: Lao People's Democratic Republic
NECHR: National Ethics Committee for Health Research
PCA: principal component analysis
OR: odds ratios
UNICEF: United Nations Children's Fund
WASH: water, sanitation and hygiene
WHO: World Health Organisation

## 1 Introduction

Acute hepatitis infection can be caused by, among others, hepatitis A virus (HAV) and hepatitis E virus (HEV), both of whichare RNA viruses, from the *Picornaviridae* and *Hepeviridae* families, respectively [1]. Unlike hepatitis B and C viruses, HAV and HEV typically do not lead to chronic liver infection and rarely fulminant hepatitis, though they can lead to symptoms such as fever, fatigue, jaundice, abdominal pain, and anorexia and can be more serious in immunocompromised individuals or pregnant women (for HEV) [1]. HAV and HEV are both primarily transmitted via the faecal‐oral route, typically through contaminated water or food [2]. HAV infection is strongly associated with unsafe water, inadequate sanitation, poor personal hygiene, and oral‐anal sex [3]. HEV can also be transmitted via contaminated drinking water and food, such as raw and undercooked animal meat, but can additionally be transmitted directly from animals such as swine [4]. In low‐ and middle‐income countries, infections are commonly linked to poor hygiene and inadequate sanitation. HAV infection is estimated to cause about 1.5 million symptomatic cases annually, with around ten million individuals experiencing asymptomatic infections [5]. Meanwhile, HEV infection is estimated to result in 20 million cases worldwide, with 3.3 million of these cases showing symptoms [6].

Adequate water, sanitation, and hygiene (WASH) are essential for human health, improving quality of life, mental well‐being, environmental protection, and reducing waterborne diseases [7]. In 2017, 90% of the global population had access to improved drinking water, whilst approximately 785 million people lacked basic water services, and 12% practiced open defecation [8]. Poor WASH has been linked with infectious diseases, particularly those transmitted by the faecal‐oral route or waterborne diseases [7]. Therefore, good WASH levels are important, including; access to improved water source such as piped water, boreholes or tube wells, protected dug wells, protected springs, and packaged or delivered water; access to improved sanitation such as pit latrines, pour‐flush latrines, ventilated pit latrines, private facilities etc; and improved hygiene practices [7].

In Southeast Asia, the Lao People's Democratic Republic (Lao PDR) has seen positive changes in access to improved drinking water sources between 1993 and 2015 [9]. Despite progress, open defecation remains a significant public health issue, with 23% of the population practicing it nationwide and higher rates in remote areas in 2017. Khammouane province, where our study was conducted, exhibited an overall open defecation rate of 29.2% in 2017 [9].

Lao PDR still continues to face a high burden of infectious diseases transmitted via the faecal‐oral route and potentially linked to poor WASH conditions. Reported outbreaks of HAV in Lao PDR include one in 2016 in Xiengkhouang province (northern Lao PDR) where more than one thousand cases were reported and confirmed by the WHO, and another in Vientiane capital in 2017 with approximately 900 cases [10]. A small serology study for anti‐HAV was conducted in rural Xiengkhouang province and urban Vientiane capital (central Lao PDR) in 2017. The results showed 62.3% anti‐HAV IgG seropositivity in Xiengkhouang province and 45.5% in Vientiane capital [10], indicating previous contact with the virus (there is no routine HAV and HEV vaccination in Lao PDR and limited/no access to voluntary vaccination [11], and antibodies are long‐lived following infection). Furthermore, anti‐HEV seroprevalence in Vientiane capital revealed that 41% of adults in contact with pigs had been exposed to HEV, compared to 18% in the control group [12]. In another study in semi‐urban central Lao PDR, exposure was potentially associated with drinking water sources, warranting further investigation [13]. Our unpublished research indicates that 84.3% and 57.9% of individuals are exposed to HAV and HEV in Oudomxay, Luangprabang, Savanakhet, Champasak provinces and Vientiane capital. These data are in line with the detection of HEV in swine [14, 15] and humans [16] in Lao PDR.

Several publications discuss infectious diseases in the Lao context, but few studies have examined WASH levels. Similarly, research in other settings rarely links WASH levels directly to exposure to waterborne infectious diseases. Therefore, we conducted this study to evaluate the WASH levels and seroprevalence of anti‐HAV and anti‐HEV in Khammouane Province, Lao PDR, and the relationship between serology and WASH levels and other variables.

## 2 Methodology

### 2.1 Study Design and Population

A cross‐sectional study was conducted from January to April 2023 in Khammouane province, central Lao PDR. The province, with a population of approximately 400,000 in 2015 across 569 villages and 10 districts [17], was chosen for its diverse ethnicities (Lao‐Loum (Lao‐Tai), Phou Thay, and Brou) [18] and their distinct cultural and WASH practices.

**Figure 1:**
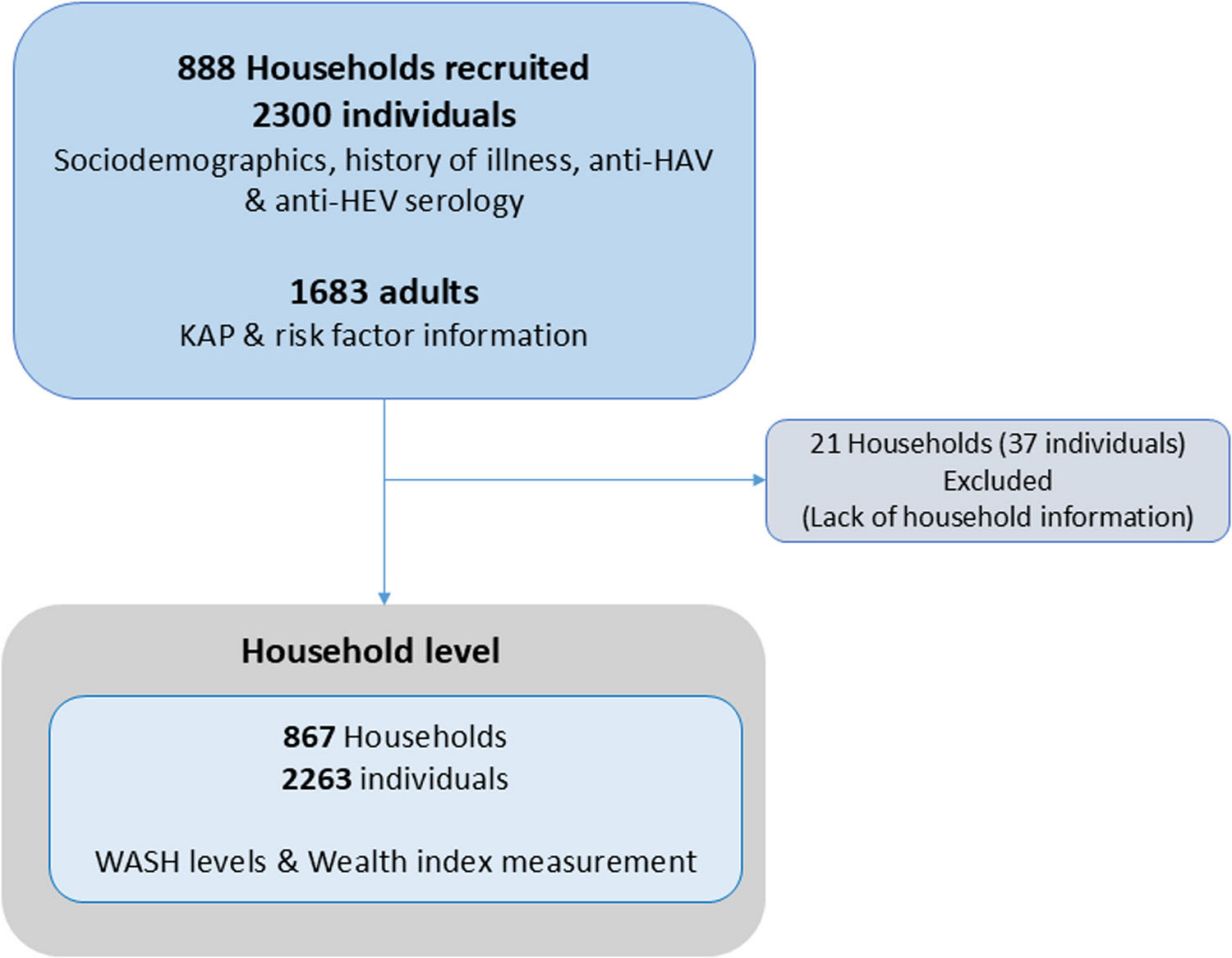
Flow chart for participant enrolment.

Cluster sampling was used to recruit the study population. Simple random sampling selected 82 out of 174 villages within 3 out of 10 districts (Figure 1). Households were randomly chosen from a compiled list of all households within a village and 30 participants per village were sampled. Investigators presented a consent form before the interviews were conducted at convenient locations such as the village office or temple. Enrolment included individuals aged 5 years and above from selected households, with consent required for those aged 18 and above and assent for those aged 15–17. Those below the age of 15 had to have parental consent. Exclusion criteria included lack of consent, refusal to answer the questionnaire, acute illness, immunological or hematologic disorders, undergoing chemotherapy, or pregnancy. This study was approved by the Lao National Ethics Committee for Health Research (NECHR) (Ref 041/2022). Each household's WASH level was assessed using a structured questionnaire based on WHO/UNICEF definitions (Table 1) [19].

**Table 1:** WASH levels scores base on UNICEF/WHO [7].

Variables	WASH levels scores				
	1	2	3	4	5
Water source service level	Surface water	Unimproved	Improved: Limited	Improved: Basic	Improved: Safely managed
Sanitation service levels	Open defecation	Unimproved	Improved: Limited	Improved: Basic	Improved: Safely managed
Hygiene service levels	No facilities	Limited	Basic	N/A	N/A

*Note:* Water source service levels: 1). Surface water: drinking water directly from a river, dam, lake, pond, stream, canal or irrigation canal; 2). Unimproved: drinking water from an unprotected dug well or unprotected spring; 3). Improved limited: drinking water from an improved source that requires a journey of more than 30 min round trip, including queuing; 4). Improved basic: < 30 min including queuing; 5). Improved safely managed: water source located on the premises, available when needed, free from faecal and priority chemical contamination. Sanitation service levels: 1). Open defecation; 2). Unimproved: Use of pit latrines without a slab or platform, hanging latrines or bucket latrines; 3). Improved limited: use of improved facilities shared between two or more households; 4). Improved basic: not shared with other households; 5). Improved safely managed: not shared with other households and excreta are safely disposed of in situ or transported and treated offsite. Hygiene service levels: 1). No facility: No handwashing facility on premises; 2). Limited: Availability of handwashing facility lacking soap and/or water at home; 3). Basic: Availability of a handwashing facility on premises with soap and water. For statistical analysis of water and sanitation levels, the classification of “improved” levels grouped “limited”, “basic” and “safely managed” together.

Data on socio‐demographics and history of illness were collected for all age groups. Participants aged 18 years and older were invited to answer a questionnaire to determine individual knowledge, risk factors, attitude and practice (KAP). WASH levels were determined per household by the head of the family.

### 2.2 Serology Assay

After completing the questionnaires, 5 mL of venous blood was collected from each participant and labelled with their ID number. The samples were centrifuged at 6000–8000 rpm for 10–15 min to separate the serum, which was temporarily stored at −20°C and later moved to a −80°C freezer for long‐term storage at Institut Pasteur du Laos. The anti‐HAV antibodies were evaluated using a competitive, qualitative‐based ELISA (Diagnostic Bioprobes, Dia‑Pro, Milan, Italy). The results were interpreted based on the Cut‐off/Sample (Co/S) values at OD = 450 nm, with a cut‐off calculated as (Positive control (PC) + Negative control (NC))/3 and were categorized as follows: < 0.9 negative, 0.9–1.1 equivocal, and > 1.1 positive [20, 21, 22]. Regarding the antibodies against HEV, an ELISA kit (DIA. PRO Diagnostic Bioprobes, Sesto San Giovanni, Milan, Italy) was used to detect anti‐HEV IgG, following the manufacturer's standard procedures. The cut‐off value was determined using the formula: Cut‐Off = NC mean OD450nm + 0.350. Results were categorized as follows: < 0.9 negative, 0.9–1.1 equivocal, and > 1.1 positive [21, 22].

### 2.3 Sample Size Calculation

The sample size for this cross‐sectional survey was calculated based on the estimated seroprevalence of anti‐HAV at different WASH levels in Khammouane province. Assuming a 1:1 ratio of high to low WASH communities, with seroprevalence rates of 35% and 50%, respectively [10], we used the Fleiss formula with continuity correction and multiplied by the design effect (DE). DE = 1 + (*n* (number of persons in each village) − 1)) x intra‐class correlation coefficient (ICC). To achieve 80% power at a 95% confidence level with an ICC of 0.238 [23], we determined that 82 clusters (villages) were needed out of 174, with 30 participants per village, totalling 2460 participants.

### 2.4 Statistical Analysis

Descriptive analysis was performed for all variables including district, age group, sex, ethnicity, education, and occupation. These were described using the number of observations (*n*), mean or median, interquartile range (IQR), and standard deviation (SD).

The wealth index measurement of households was assessed using principal component analysis (PCA), based on guidelines from the Health Nutrition and Population/World Bank in 2000 [24]. Briefly, PCA was performed on a range of household assets, including housing characteristics (materials used for the roof, floor, and walls), access to electricity, and ownership of items such as a television, radio, refrigerator, car, bicycle, and motorcycle. Land use and ownership of livestock (buffalo, cow, pig, goat, and poultry) were also included. Based on the PCA results, wealth index measurement was stratified into three wealth tertiles: poor, middle, and high.

Bivariate and multivariable analysis of anti‐HAV and anti‐HEV antibodies were performed, and the associations with socio‐demographic and risk factors were expressed as odds ratios (OR) and adjusted odds ratios (aOR) with 95% confidence intervals (CI). The variables included in the multivariable analysis for anti‐HAV comprised socio‐demographic factors such as district, age, sex, education, occupation, wealth index measurement, WASH levels, and risk factors such as consuming undercooked meat, leftover food, and raw snails. For anti‐HEV, similar socio‐demographic factors were considered, along with risk factors like consuming raw meat, domestic animals, pig feeding, and pig slaughter. Variables were included in anti‐HAV or anti‐HEV final models if a low Akaike information criterion score was shown. We performed a logistic regression model using Generalized Estimating Equations to account for the clustering of participants within villages. The predicted probabilities of seropositivity across age were estimated using the marginal effects after logistic regression and the ages at which 50% of the population were anti‐HAV and anti‐HEV IgG seropositive were estimated and compared. *p* ≤ 0.05 was considered to be significant.

## 3 Results

### 3.1 Socio‐Demographics

Two of the selected villages were excluded as they did not wish to participate and we were able to randomize one further village to compensate. Therefore, serum samples were collected from 2300 participants in 81 villages from the three selected districts: Nakai (349; 15.2%), Mahaxay (1047; 45.5%), and Bualapha (904; 39.3%). Less than half of the participants were male (1033; 44.9%) and the average age was 32.0 years ( ± 16.6), ranging from 5 to 87 years. The majority were Lao‐Loum ethnicity (588; 25.6%), follow by Makong (510; 22.2%) and Phuthai (507; 22.0%). Education levels of adult participants (*n* = 1683) varied: 561 (33.3%) had not received education, approximately half completed primary school, and only 39 (2.3%) attained a university degree. The majority of adults were engaged in farming (1534; 91.2%). In all participants with available data (*n* = 2263), wealth index measurement was stratified into three categories: poor (754; 33.3%), moderate (753; 33.3%), and high (756; 33.4%) (Table 2).

**Table 2:** Socio‐demographics.

	Total
Variables	*N* = 2300 (%)
District	
*Nakaiy*	349 (15.2)
*Mahaxay*	1047 (45.5)
*Bualapha*	904 (39.3)
Sex	
*Male*	1033 (44.9)
*Female*	1267 (55.1)
Mean age in years, (SD)	32.0 ( ± 16.6)
Age group	
≤ *10 yrs*	229 (10.0)
*11–20 yrs*	471 (20.5)
*21–30 yrs*	368 (16.0)
*31–40 yrs*	526 (22.9)
*41–50 yrs*	369 (16.0)
> *50 yrs*	337 (14.7)
Ethnicity	
*Lao‐Loum*	588 (25.6)
*Makong*	510 (22.2)
*Bru*	20 (0.9)
*Phouthai*	507 (22.0)
*Kalerng*	148 (6.4)
*Guan*	17 (0.7)
*Jalee*	178 (7.7)
*Sum*	80 (3.5)
*Tri*	104 (4.5)
*Others*	148 (6.4)
Levels of education ( ≥ 18 years old; *n* = 1683)	
*No schooling*	561 (33.3)
*Primary school*	659 (39.2)
*Lower 2nd school*	146 (8.7)
*Upper 2nd school*	278 (16.5)
*University*	39 (2.3)
Occupation, ( ≥ 18 years old; *n* = 1683)	
*Student*	15 (0.9)
*Farmers/house wife*	1534 (91.2)
*Office staff/commerce/business*	134 (7.9)
Wealth index measurement, (*n* = 2263)	
*Poor tertile*	754 (33.3)
*Middle tertile*	753 (33.3)
*High tertile*	756 (33.4)

### 3.2 WASH Levels Assessment

The WASH assessment results show a wide range of levels (Table 3).

**Table 3:** WASH assessment levels, *n* = 2263.

	Total
Variables	*N* = 2263 (%)
Water assessment level	
*Surface water*	272 (12.0)
*Unimproved*	89 (3.9)
*Improved* *	1902 (84.0)
Sanitation assessment level	
*Open defecation*	497 (22.0)
*Unimproved*	0 (0.0)
*Improved* **	1766 (78.0)
Hygiene assessment level	
*No facility*	210 (9.3)
*Limited*	540 (23.9)
*Basic*	1513 (66.9)

*Note:* For definitions of the different WASH levels, see Table 1.

* For water level assessment,” Improved” levels grouped “limited”, “basic”, and “safely managed” together.

** For sanitation level assessment,” Improved” levels grouped “limited”, “basic”, and “safely managed” together.

### 3.3 Anti‐HAV Seroprevalence and Associated Variables

**Figure 2:**
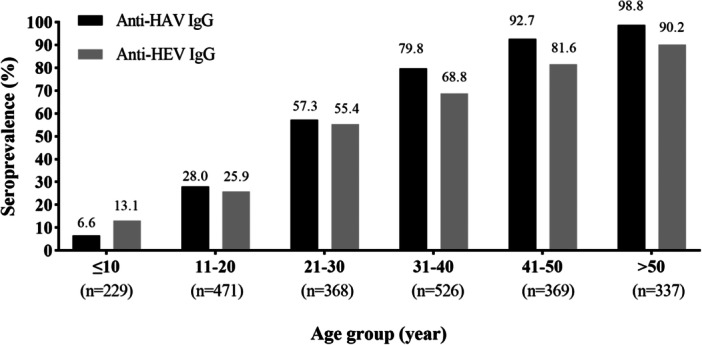
Seroprevalence of anti‐HAV and anti‐HEV IgG by age group. Numbers in brackets represent the number of participants per age group.

**Figure 3:**
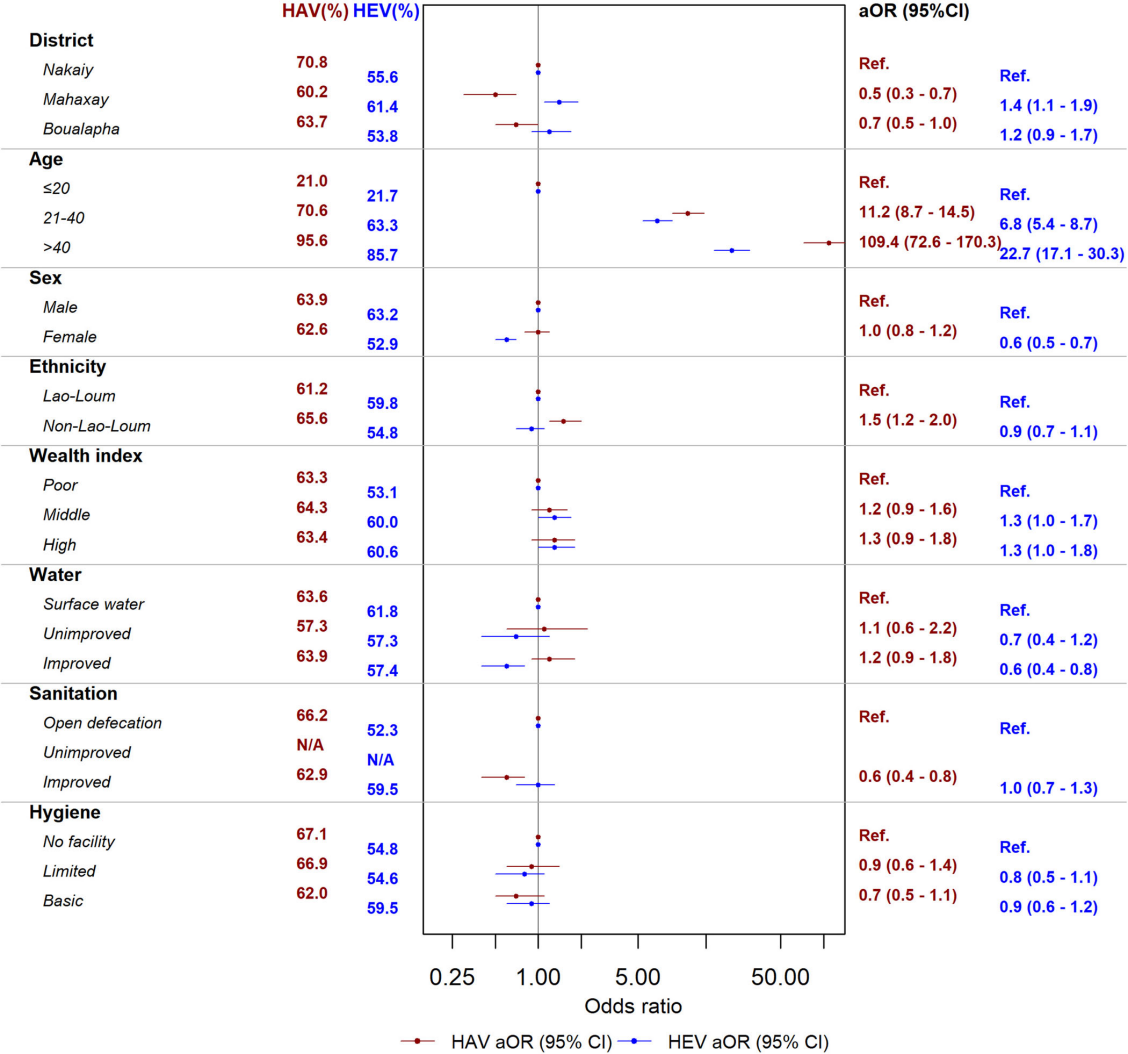
Multivariable analysis of anti‐HAV and anti‐HEV IgG seroprevalence in all participants (*n* = 2300).

**Figure 4:**
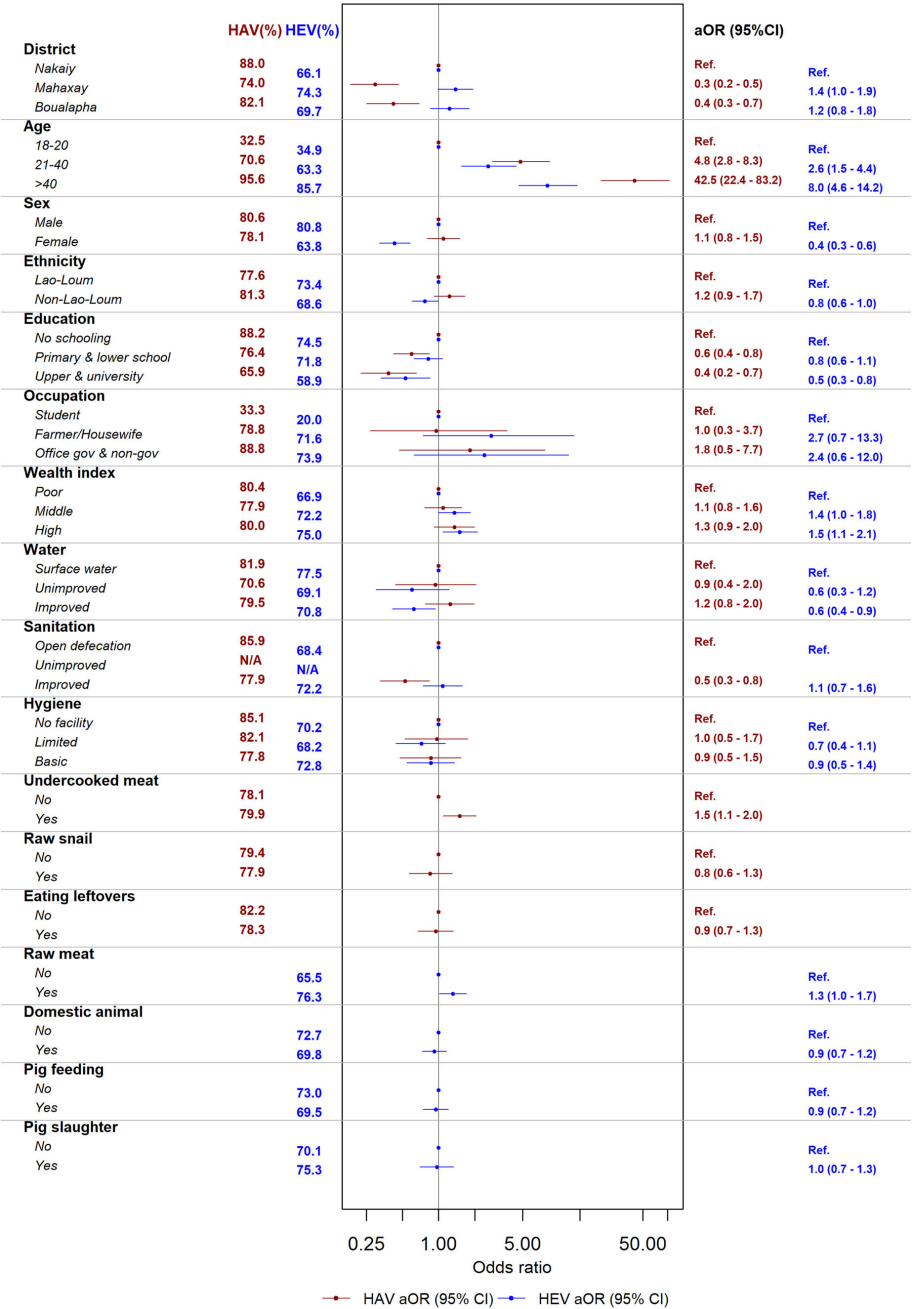
Multivariable analysis of anti‐HAV and anti‐HEV IgG seroprevalence in adults aged ≥ 18 (*n* = 1683).

**Figure 5:**
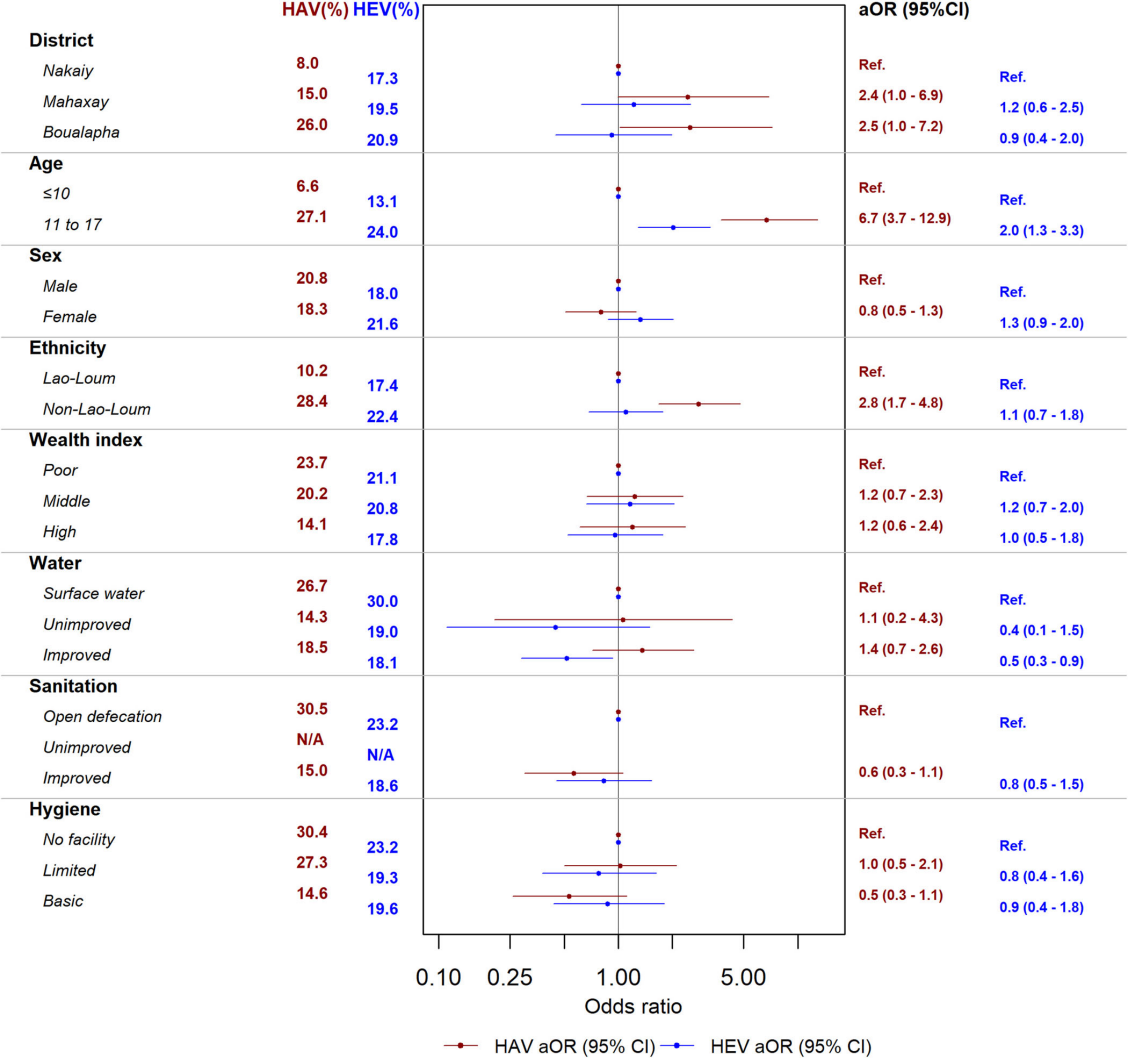
Multivariable analysis of anti‐HAV and anti‐HEV IgG seroprevalence in child participants (aged < 18 years; *n* = 617).

In total, 1453/2300 (63.2%) participants were anti‐HAV seropositive (Figure 2). Improved sanitation (limited, basic, and safely managed) was associated with a lower odds of being anti‐HAV seropositive overall compared to open defecation (aOR = 0.6, 95% CI = 0.4–0.8, *p* = 0.001) (Figure 3, Table S1), with similar associations in adults and children (Figures 4 and 5, Tables S3 and S5). However, we observed no significant association with the water source. After multivariable analysis, compared to those under the age of 21 years, seroprevalence was higher in those aged 21–40 years (aOR = 11.2, 95%CI = 8.7–14.5; *p* < 0.001) and those aged greater than 40 years (aOR = 109.4, 95%CI = 72.6–170.3; *p* < 0.001). There was variation of overall anti‐HAV seroprevalence by district: Mahaxay and Bualapha showed a significantly lower seroprevalence of anti‐HAV compared to Nakaiy district (aOR = 0.5, 95%CI = 0.3–0.7; *p* < 0.001 and aOR = 0.7, 95%CI = 0.5–1.0, *p* = 0.04, respectively). Overall, the non‐Lao‐Loum group had a significantly higher likelihood of anti‐HAV seropositivity compared to the Lao‐Loum ethnic group (aOR = 1.5, 95%CI = 1.2–2.0, *p* = 0.001), with a similar pattern in children as in adults (Figures 3 and 5, Tables S1 and S5).

Those adults completing upper school or university showed lower odds of anti‐HAV seropositivity compared to those with no schooling (aOR = 0.6, 95% CI = 0.4–0.8, *p* = 0.003 and aOR = 0.4, 95% CI = 0.2–0.7, *p* < 0.001). Consumption of undercooked meat was a significant risk factor for anti‐HAV seropositivity in adults (aOR = 1.5, 95% CI = 1.1–2.0, *p* = 0.01) (Figure 4, Table S3).

### 3.4 Anti‐HEV Seroprevalence and Associated Demographic Variables

In total, 1323/2300 (57.5%) participants were anti‐HEV seropositive (Figure 2). For water assessment levels, compared to surface water as source, improved water sources (limited, basic, and improved) were associated with a lower probability of being anti‐HEV seropositive (aOR = 0.6, 95% CI = 0.4–0.8, *p* = 0.002) (Figure 3, Table S2) and similar associations were found in adults and children (Figures 4 and 5 and Tables S4 and S6). Compared to those under the age of 21 years seroprevalence was significantly higher in those aged 21–40 years (aOR = 6.8, 95%CI = 5.4–8.7; *p* < 0.001) and those aged more than 40 years (aOR = 22.7, 95%CI = 17.1–30.3; *p* < 0.001) (Figure 3 and Table S2). Mahaxay demonstrated a statistically significantly higher likelihood of anti‐HEV compared to Nakaiy (aOR = 1.4, 95% CI = 1.1–1.9, *p* = 0.02). Females showed a significantly lower seroprevalence compared to males (aOR = 0.6, 95% CI = 0.5–0.7, *p* < 0.001), a difference that was seen in adults but not in the children (Figures 4 and 5 and Tables S4 and S6). High income (aOR = 1.3, 95% CI = 1.0–1.8, *p* = 0.05) showed a significantly higher seroprevalence compared to poor income.

In adults, non‐Lao‐Loum ethnic groups had a significantly lower odds of HEV seropositivity (aOR = 0.8, 95% CI = 0.6–1.0, *p* = 0.04) compared to Lao‐Loum ethnicity. Adults having attended upper school or university were less likely anti‐HEV IgG positive as compared to adults without schooling (aOR = 0.5, 95% CI = 0.3–0.9, *p* = 0.01). Consumption of raw meat by adults was a significant risk factor (aOR = 1.3, 95% CI = 1.0–1.7, *p* = 0.04) (Figure 4 and Table S4).

### 3.5 Seroprevalence of Anti‐HAV and Anti‐HEV at Different Wash Levels

**Figure 6:**
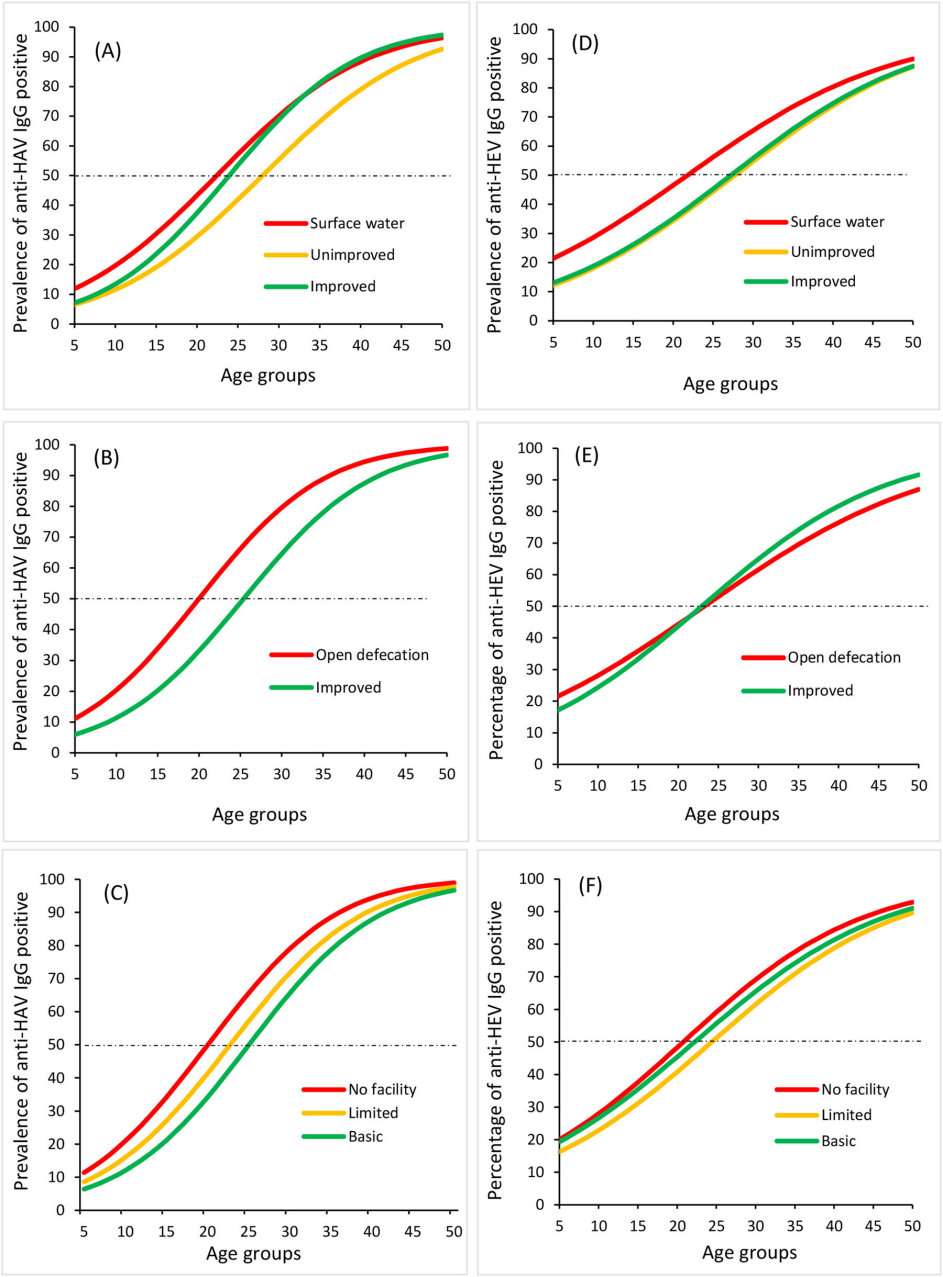
Age‐stratified anti‐HAV and anti‐HEV seroprevalence estimates according to; water assessment level (A and D); sanitation assessment level (B and E); hygiene assessment level (C and F).

Overall, the predicted mean age at which the probability of being infected is 50% was 24 and 27 years old for anti‐HAV and anti‐HEV, respectively. For anti‐HAV antibodies, the predicted mean age was significantly higher for those with improved sanitation compared to open defecation (*p* = 0.0001), and for those with basic hygiene compared with no facility (*p* = 0.01). None of the other trends reached statistical significance (Figure 6).

## 4 Discussion

Our data indicate that 12% of the study population continues to rely on surface water sources, 22% still engage in open defecation, and 9.3% lack hygiene facilities within their households. These findings are generally consistent with the Lao Social Indicator Survey (LSIS) from 2017 when 12.9% of participants from Khammouane province reported to rely on surface water sources, 29.2% practiced open defecation, and 23.1% lacked hygiene facilities [9]. The difference in hygiene levels between our study in 2023 and those from LSIS in 2017 may relate to a difference in sampling methods and choice of location, rather than any real decrease in hygiene levels. Both surveys reflect that despite overall improvements in WASH infrastructure, significant gaps remain.

We found a high seroprevalence of anti‐HAV at 63.2% and anti‐HEV at 57.5% overall. These results were higher than reports from other low‐ to middle‐income countries (sub‐Saharan Africa, India, Thailand at 30%–40% [25, 26, 27] and Burkina Faso at 15%–20% [21], respectively), and aligned with the trend of high seroprevalence, particularly in older age groups as exposure to the viruses accumulates over time due to ongoing transmission. In addition, the young age groups showed significant exposure, possibly reflecting poor WASH and frequent contact with animals such as swine in the case of HEV (genotype 3 and 4) [28]. The HAV data are similar to our previous study in rural Xiengkhouang in northern Lao PDR, which had slightly higher anti‐HAV seroprevalence in the younger age groups, probably related to a recorded HAV outbreak shortly before participant recruitment [10].

Anti‐HAV seroprevalence was significantly lower in those with improved sanitation than those practicing open defecation and anti‐HEV seroprevalence was lower in those with improved water source than those using surface water. Comparing the ages at which 50% of the study populations are estimated to be seropositive for anti‐HAV or anti‐HEV antibodies can give another epidemiological snapshot and confirmation of the exposure levels in different settings. When this age is low, it indicates increased exposure to the viruses. For anti‐HAV antibodies, this age was significantly higher for those with improved sanitation compared to open defecation, and for those with basic hygiene compared with no facility. These data emphasise that improving water sources, sanitation levels, and hygiene facilities can reduce exposure to HAV and HEV, although a larger sample size and more disparate populations would be needed to see more highly significant trends. Indeed, other studies showed that individuals in settings with poor sanitation and water quality are exposed to hepatitis A at younger ages than those in regions with better infrastructure and improvements in WASH have been linked to shifts in the epidemiological curve as we see in the current study [26, 29].

Anti‐HAV seroprevalence was significantly higher in the non‐Lao‐Loum groups (both children and adults), suggesting more frequent exposure. This may reflect the socioeconomic and WASH level disparities between different ethnicities or different WASH practices, which have been shown in other studies [9]. There was an opposite trend for anti‐HEV, perhaps due to unknown risk practices and different routes of transmission, which requires further investigation. We saw differences in anti‐HAV and anti‐HEV seroprevalence between the different districts, which again could reflect the different socioeconomic status or risk practices.

We observed higher anti‐HEV seroprevalence in those that reported eating raw meat, which has been previously identified as a major route of HEV [30] transmission. Similarly, adults consuming undercooked meat had a higher anti‐HAV seroprevalence. Education was also significantly associated with seroprevalence of anti‐HAV and anti‐HEV. Importantly, there are likely to be specific risk factors that are not covered in this study, such as historic WASH levels which will impact on exposure.

Interestingly, males had a higher seroprevalence of anti‐HEV than females – a difference that was not seen for anti‐HAV seroprevalence. The reasons are not fully understood but this might be a consequence of higher contact of males with swine such as pig rearing, pig feeding and/or swine slaughtering and behaviours of males such as, consumption of undercooked pork or engagement in high‐risk occupational activities. The higher HEV seroprevalence among males has also been observed in other studies [31].

Our study had several limitations. Firstly, the geographical proximity of the three selected districts may have limited the diversity of environmental and socioeconomic conditions, as all the three were rural districts. Furthermore, it might also limit the generalizability of our findings to the rest of the province and other provinces of the country. However, our study sites are a good representation of economic and environmental conditions of rural districts of Lao PDR. Similarly, logistical challenges, including limited accessibility and security concerns, prevented us from sampling certain very remote areas, which may have resulted in under‐representation of these regions in our analysis. Importantly, the absence of detailed timelines for WASH infrastructure improvements limits the interpretation of the results because the timing of these interventions is critical to understanding their impact on the age‐stratified exposure. We also cannot be sure as to the relative importance of the different transmission routes, for example, WASH‐related versus zoonotic. Finally, the reliance on self‐reported data through interviews for WASH assessments, without direct observational validation, may have introduced reporting bias and may thus have slightly affected the reliability of our WASH‐related findings. Nevertheless, this study provided important and novel insights into the population exposure to HAV and HEV and WASH conditions in this area.

The finding that approximately 50% of young adults are seronegative for anti‐HAV and anti‐HEV reflects an overall improvement in hygiene and living conditions but also suggests that a substantial proportion of the population remains at risk of severe disease. Indeed, the study population may be classified as having ‘low to intermediate’ HAV endemicity [32]. As observed in several lower‐middle income countries [5], the transition from high to lower endemicity can lead to a shift in disease burden, with hepatitis A becoming a leading cause of acute hepatitis requiring liver transplantation. Therefore, consideration should be given to the implementation of routine childhood HAV vaccination, as recommended by the WHO [5]. HEV vaccine, on the other hand, is not licenced in many countries. It is possible that it could be used to protect pregnant women in outbreak situations [33] but its introduction into the routine vaccination schedule is not recommended.

## 5 Conclusion

Our findings show that WASH levels in Khammouane province remain low and that previous exposure to HAV and HEV is high. Exposure to HAV was associated with ethnicity, district, consumption of undercooked meat and sanitation level, whilst HEV exposure was linked with sex, water source, and raw meat consumption. These risk factors could be addressed by improving WASH and raising awareness regarding faecal oral infections and consideration of HAV vaccination in children.

## Author Contributions

Conceptualization, Vilaysone Khounvisith, Antony P. Black, Judith M. Hübschen, and Peter Odermatt; methodology, Vilaysone Khounvisith, Siriphone Virachith, and Antony P. Black, Jan Hattendoft; formal analysis, Vilaysone Khounvisith, Antony P. Black and Peter Odermatt, Jan Hattendoft; investigation, Vilaysone Khounvisith, Siriphone Virachith, Latdavone Khenkha, Bounta Vongphachanh and Nouna Innoula; writing – original draft, Vilaysone Khounvisith, and Antony P. Black; writing – review and editing, Vilaysone Khounvisith, and Antony P. Black, Judith M. Hübschen and Peter Odermatt; supervision, Peter Odermatt, Antony P. Black and Judith M. Hübschen and funding acquisition, Judith M.H. Hübschen, Antony P. Black. All authors have read and agreed to the published version of the manuscript.

## Ethics Statement

Ethics approval was obtained from the Lao National Ethics Committee for Health Research (NECHR), Ministry of Health (MoH), Vientiane, Lao PDR (Ref. no.041/2022 NECHR). Informed consent was obtained from all parents or legal guardians for subjects under 15 years of age, with assent forms obtained for participants aged 15 to 17, while individuals aged 18 years and older provided consent independently.

## Conflicts of Interest

The authors declare no conflicts of interest.

## Supporting information


**Table S1:** Bivariate and multivariable analysis of associations between seroprevalence of anti‐HAV, socio‐2 demographics and WASH levels in all age groups. **Table S2:** Bivariate and multivariable analysis of associations between seroprevalence of anti‐HEV, socio‐7 demographics and WASH levels in all age‐groups. **Table S3:** Bivariate and multivariable analysis of associations between seroprevalence of anti‐HAV, socio‐15 demographics, WASH levels and risk factors in participants aged 18 years old and above. **Table S4:** Bivariate and multivariable analysis of associations between seroprevalence of anti‐HEV, socio‐20 demographics, WASH levels and risk factors in participants aged 18 years old and above. **Table S5:** Bivariate and multivariable analysis of associations between seroprevalence of anti‐HAV, socio‐29 demographics and WASH levels in participants aged less than 18 years old. **Table S6:** Bivariate and multivariable analysis of associations between seroprevalence of anti‐HEV, socio‐34 demographics and WASH levels in participants aged less than 18 years old.

## Data Availability

The data that support the findings of this study are available on request from the corresponding author. The data are not publicly available due to privacy or ethical restrictions. The datasets used and/or analysed during this study are a vailable from the corresponding author on reasonable request.
