# Supplementary material for: Hepatitis A and E Virus Seroprevalence and Water, Sanitation and Hygiene Levels in Rural Areas of Khammouane Province, Lao People's Democratic Republic: A Cross‐Sectional Study
Source: J Med Virol. 2025 Jul 30;97(8):e70524. doi: 10.1002/jmv.70524 (PMC12308774; doi:10.1002/jmv.70524)
Supplement: Supplementary file 1 — Table S1: Bivariate and multivariable analysis of associations between seroprevalence of anti‐HAV, socio‐2 demographics and WASH levels in all age groups. Table S2: Bivariate and multivariable analysis of associations between seroprevalence of anti‐HEV, socio‐7 demographics and WASH levels in all age‐groups. Table S3: Bivariate and multivariable analysis of associations between seroprevalence of anti‐HAV, socio‐15 demographics, WASH levels and risk factors in participants aged 18 years old and above. Table S4: Bivariate and multivariable analysis of associations between seroprevalence of anti‐HEV, socio‐20 demographics, WASH levels and risk factors in participants aged 18 years old and above. Table S5: Bivariate and multivariable analysis of associations between seroprevalence of anti‐HAV, socio‐29 demographics and WASH levels in participants aged less than 18 years old. Table S6: Bivariate and multivariable analysis of associations between seroprevalence of anti‐HEV, socio‐34 demographics and WASH levels in participants aged less than 18 years old. [file JMV-97-e70524-s001.pdf]

Supplementary data

Table S1 Bivariate and multivariable analysis of associations between seroprevalence of anti-HAV, socio-demographics and WASH levels in all age groups.

	Anti-HAV seroprevalence							
	Bivariate analysis					Multivariable analysis		
Variables	n/n total	% positive	OR	95% CI	p-value	OR	95% CI	p-value
District, n=2300								
Nakaiy	247/349	70.8	Ref			Ref		
Mahaxay	630/1047	60.2	0.6	0.5-0.8	<0.001	0.5	0.3-0.7	<0.001
Bualapha	576/904	63.7	0.7	0.6-0.9	0.02	0.7	0.5-1.0	0.04
Sex, n=2300								
Male	660/1033	63.9	Ref			Ref		
Female	793/1267	62.6	0.9	0.8-1.1	0.52	1.0	0.8-1.2	0.95
Age group, n=2300								
5-20	147/700	21.0	Ref			Ref		
21-40	631/894	70.6	9.0	7.1-11.4	<0.001	11.2	8.7-14.5	<0.001
>40	675/706	95.6	81.9	54.7-122.6	<0.001	109.4	72.6-170.3	<0.001
Ethnicity, n=2300								
Lao-Tai	764/1249	61.2	Ref			Ref		
Non-Lao-Tai	689/1051	65.6	1.2	1.0-1.4	0.03	1.5	1.2-2.0	0.001
Wealth index measurement, n=2263								
Poor	477/754	63.3	Ref			Ref		
Middle	484/753	64.3	1.0	0.8-1.3	0.68	1.2	0.9-1.6	0.31
High	479/756	63.4	1.0	0.8-1.2	0.97	1.3	0.9-1.8	0.11
Water assessment levels, n=2263								
Surface water	173/272	63.6	Ref			Ref		
Unimproved water	51/89	57.3	0.8	0.5-1.3	0.29	1.1	0.6-2.2	0.75
Improved water	1216/1902	63.9	1.0	0.8-1.3	0.92	1.2	0.9-1.8	0.26
Sanitation assessment levels, n=2263								
Open defecation	329/497	66.2	Ref			Ref		
Improved Sanitation	1111/1766	62.9	0.9	0.7-1.1	0.18	0.6	0.4-0.8	0.001
Hygiene assessment levels, n=2263								
No facilities	141/210	67.1	Ref			Ref		
Limited	361/540	66.9	1.0	0.7-1.1	0.94	0.9	0.6-1.4	0.72
Basic	938/1513	62.0	0.8	0.6-1.1	0.15	0.7	0.5-1.1	0.11

Table S2 Bivariate and multivariable analysis of associations between seroprevalence of anti-HEV, socio-demographics and WASH levels in all age-groups.

			Anti-HEV seroprevalence					
			Bivariate analysis			Multivariable analysis		
Variables	n/n total	% positive	OR	95% CI	p-value	OR	95% CI	p-value
District, n=2300								
Nakaiy	194/349	55.6	Ref			Ref		
Mahaxay	643/1047	61.4	1.3	1.0-1.6	0.06	1.4	1.1-1.9	0.02
Bualapha	486/904	53.8	0.9	0.7-1.2	0.56	1.2	0.9-1.7	0.23
Sex, n=2300								
Male	653/1033	63.2	Ref			Ref		
Female	670/1267	52.9	0.7	0.6-0.8	<0.001	0.6	0.5-0.7	<0.001
Age group, n=2300								
5-20	152/700	21.7	Ref			Ref		
21-40	566/894	63.3	6.2	5.0-8.0	<0.001	6.8	5.4-8.7	<0.001
>40	605/706	85.7	21.6	16.4-28.5	<0.001	22.7	17.1-30.3	<0.001
Ethnicity, n=2300								
Lao-Tai	747/1249	59.8	Ref			Ref		
Non-Lao-Tai	567/1051	54.8	0.8	0.7-1.0	0.02	0.9	0.7-1.1	0.19
Wealth index measurement, n=2263								
Poor	400/754	53.1	Ref			Ref		
Middle	452/753	60.0	1.3	1.1-1.6	0.006	1.3	1.0-1.7	0.05
High	485/756	60.6	1.4	1.1-1.7	0.003	1.3	1.0-1.8	0.05
Water assessment levels, n=2263								
Surface water	168/272	61.8	Ref			Ref		
Unimproved water	51/89	57.3	0.8	0.5-1.4	0.46	0.7	0.4-1.2	0.16
Improved water	1091/1902	57.4	0.8	0.6-1.1	0.17	0.6	0.4-0.8	0.002
Sanitation assessment levels, n=2263								
Open defecation	260/497	52.3	Ref			Ref		
Improved Sanitation	1050/1766	59.5	1.3	1.1-1.7	0.004	1.0	0.7-1.3	0.82
Hygiene assessment levels, n=2263								
No facilities	115/210	54.8	Ref			Ref		
Limited	295/540	54.6	1.0	0.7-1.4	0.97	0.8	0.5-1.1	0.19
Basic	900/1513	59.5	1.2	0.9-1.6	0.19	0.9	0.6-1.2	0.40

Table S3 Bivariate and multivariable analysis of associations between seroprevalence of anti-HAV, socio-demographics, WASH levels and risk factors in participants aged 18 years old and above.

		**Anti-HAV seroprevalence**							
		Bivariate analysis					Multivariable analysis		
Variables		n/n total	% positive	OR	95% CI	p-value	OR	95% CI	p-value
District, n=1683									
	Nakaiy	241/274	88.0	Ref			Ref		
	Mahaxay	593/801	74.0	0.4	0.3-0.6	<0.001	0.3	0.2-0.5	<0.001
	Bualapha	499/608	82.1	0.6	0.4-1.0	0.03	0.4	0.3-0.7	0.001
Sex, n=1683									
	Male	600/744	80.6	Ref			Ref		
	Female	733/939	78.1	0.9	0.7-1.1	0.20	1.1	0.8-1.5	0.56
Age group, n=1683									
	18-20	27/83	32.5	Ref			Ref		
	21-40	631/894	70.6	5.0	3.1-8.1	<0.001	4.8	2.8-8.3	<0.001
	>40	675/706	95.6	45.3	25.2-81.1	<0.001	42.5	22.4-83.2	<0.001
Ethnicity, n=1683									
	Lao-Loum	733/945	77.6	Ref			Ref		
	Non-Lao-Loum	600/738	81.3	1.3	1.0-1.6	0.06	1.2	0.9-1.7	0.18
Levels of education completed, n=1683									
	No school	495/561	88.2	Ref			Ref		
	Primary & lower school	716/937	76.4	0.4	0.3-0.6	<0.001	0.6	0.4-0.8	0.003
	Upper & university	122/185	65.9	0.3	0.2-0.4	<0.001	0.4	0.2-0.7	<0.001
Occupation, n=1683									
	Student	05/15	33.3	Ref			Ref		
	Farmer/housewife	1209/1534	78.8	7.4	2.3-21.9	<0.001	1.0	0.3-3.7	0.95
	Office staff/Commerce/Business	119/134	88.8	15.9	4.8-52.7	<0.001	1.8	0.5-7.7	0.40
Wealth index measurement, n=1666									
	Poor tertile	423/526	80.4	Ref			Ref		
	Middle tertile	448/575	77.9	0.9	0.6-1.2	0.31	1.1	0.8-1.6	0.64
	High tertile	452/565	80.0	1.0	0.7-1.3	0.86	1.3	0.9-2.0	0.13
Water assessment levels, n=1666									
	Surface water	149/182	81.9	Ref			Ref		
	Unimproved water	48/68	70.6	0.5	0.3-1.0	0.05	0.9	0.4-2.0	0.87
	Limited, Basic & Improved water	1126/1416	79.5	0.9	0.6-1.3	0.46	1.2	0.8-2.0	0.36
Sanitation assessment levels, n=1666									
	Open defecation	275/320	85.9	Ref			Ref		
	Limited, Basic & Improved sanitation	1048/1346	77.9	0.6	0.4-0.8	0.001	0.5	0.3-0.8	0.008
Hygiene assessment levels, n=1666									
	No facilities	120/141	85.1	Ref			Ref		

Limited	320/390	82.1	0.8	0.5-1.4	0.41	1.0	0.5-1.7	0.92
Basic	883/1135	77.8	0.6	0.4-1.0	0.05	0.9	0.5-1.5	0.62
Consume undercooked meat, n=1683								
No	525/672	78.1	Ref			Ref		
yes	808/2011	79.9	1.1	0.9-1.4	0.37	1.5	1.1-2.0	0.01
Consume raw snail, n=1683								
No	1153/1452	79.4	Ref			Ref		
Yes	180/231	77.9	0.9	0.7-1.3	0.61	0.8	0.6-1.3	0.75
Consume remaining food, n=1683								
No	324/394	82.2	Ref			Ref		
Yes	1009/1289	78.3	0.8	0.6-1.0	0.09	0.9	0.7-1.3	0.44

Table S4 Bivariate and multivariable analysis of associations between seroprevalence of anti-HEV, socio-demographics, WASH levels and risk factors in participants aged 18 years old and above.

	Anti-HEV seroprevalence							
		Bivariate analysis				Multivariable analysis		
Variables	n/n total	% positive	OR	95% CI	p-value	OR	95% CI	p-value
District, n=1683								
Nakaiy	181/274	66.1	Ref			Ref		
Mahaxay	595/801	74.3	1.5	1.1-2.0	0.01	1.4	1.0-1.9	0.07
Bualapha	424/608	69.7	1.2	0.9-1.6	0.28	1.2	0.8-1.8	0.28
Sex, n=1683								
Male	601/744	80.8	Ref			Ref		
Female	599/939	63.8	0.4	0.3-0.5	<0.001	0.4	0.3-0.6	<0.001
Age group, n=1683								
18- 20	29/83	34.9	Ref			Ref		
21-40	566/894	63.3	3.2	2.0-5.2	<0.001	2.6	1.5-4.4	<0.001
>40	605/706	85.7	11.2	6.8-18.4	<0.001	8.0	4.6-14.2	<0.001
Ethnicity, n=1683								
Lao-Loum	694/945	73.4	Ref			Ref		
Non-Lao-Loum	506/738	68.6	0.8	0.6-1.0	0.03	0.8	0.6-1.0	0.04
Levels of education completed, n=1683								
No school	418/561	74.5	Ref			Ref		
Primary & lower school	673/937	71.8	0.4	0.3-0.6	<0.001	0.8	0.6-1.1	0.16
Upper & university	109/185	58.9	0.3	0.2-0.4	<0.001	0.5	0.3-0.8	0.01
Occupation, n=1683								
Student	3/15	20.0	Ref			Ref		
Farmer/housewife	1098/1534	71.6	10.1	2.8-35.9	<0.001	2.7	0.7-13.3	0.16
Office staff/Commerce/Business	99/134	73.9	11.3	3.0-42.5	<0.001	2.4	0.6-12.0	0.23

Wealth index measurement, n=1666								
Poor tertile	352/526	66.9	Ref			Ref		
Middle tertile	415/575	72.2	1.3	1.0-1.7	0.06	1.4	1.0-1.8	0.06
High tertile	424/565	75.0	1.5	1.1-1.9	0.003	1.5	1.1-2.1	0.02
Water assessment levels, n=1666								
Surface water	141/182	77.5	Ref			Ref		
Unimproved water	47/68	69.1	0.7	0.3-1.2	0.18	0.6	0.3-1.2	0.15
Limited, Basic & Improved water	1003/1416	70.8	0.7	0.5-1.0	0.06	0.6	0.4-0.9	0.03
Sanitation assessment levels, n=1666								
Open defecation	219/320	68.4	Ref			Ref		
Limited, Basic & Improved sanitation	972/1346	72.2	1.2	0.9-1.6	0.18	1.1	0.7-1.6	0.70
Hygiene assessment levels, n=1666								
No facilities	99/141	70.2	Ref			Ref		
Limited	266/390	68.2	0.9	0.6-1.4	0.66	0.7	0.4-1.1	0.17
Basic	826/1135	72.8	1.1	0.8-1.7	0.52	0.9	0.5-1.4	0.52
Consume raw meat, n=1683								
No	510/779	65.5	Ref			Ref		
Yes	690/904	76.3	1.7	1.4-2.1	<0.001	1.3	1.0-1.7	0.04
Domestic animal, n=1683								
No	632/869	72.7	Ref			Ref		
Yes	568/814	69.8	0.9	0.7-1.1	0.18	0.9	0.7-1.2	0.48
Pig feeding n=1683								
No	643/881	73.0	Ref			Ref		
Yes	557/802	69.5	0.8	0.7-1.0	0.11	0.9	0.7-1.2	0.64
Pig slaughter								
No	908/1295	70.1	Ref			Ref		
Yes	292/388	75.3	1.3	1.0-1.7	0.05	1.0	0.7-1.3	0.84

Table S5 Bivariate and multivariable analysis of associations between seroprevalence of anti-HAV, socio-demographics and WASH levels in participants aged less than 18 years old.

	Anti-HAV seroprevalence							
	Bivariate analysis					Multivariable analysis		
Variables	n/n total	% positive	OR	95% CI	p-value	OR	95% CI	p-value
District, n=617								
Nakaiy	06/75	8.0	Ref			Ref		
Mahaxay	37/246	15.0	2.0	0.8-5.0	0.21	2.4	1.0-6.9	0.07
Bualapha	77/296	26.0	4.0	1.7-9.7	0.002	2.5	1.0-7.2	0.06
Sex, n=617								
Male	60/289	20.8	Ref			Ref		
Female	60/328	18.3	0.9	0.6-1.3	0.44	0.8	0.5-1.3	0.59
Age group, n=617								
5-10	15/229	6.6	Ref			Ref		
11-17	105/388	27.1	5.3	3.0-9.4	<0.001	6.7	3.7-12.9	<0.001
Ethnicity, n=617								
Lao-Tai	31/304	10.2	Ref			Ref		
Non-Lao-Tai	89/313	28.4	3.5	2.2-5.5	<0.001	2.8	1.7-4.8	<0.001
Wealth index measurement, n=597								
Poor tertile	54/228	23.7	Ref			Ref		
Middle tertile	36/178	20.2	0.8	0.5-1.3	0.41	1.2	0.7-2.3	0.50
High tertile	27/191	14.1	0.5	0.3-0.9	0.02	1.2	0.6-2.4	0.60
Water assessment levels, n=597								
Surface water	24/90	26.7	Ref			Ref		
Unimproved water	03/21	14.3	0.5	0.1-1.7	0.24	1.1	0.2-4.3	0.93
Limited, Basic & Improved water	90/486	18.5	0.6	0.4-1.1	0.08	1.4	0.7-2.6	0.35
Sanitation assessment levels, n=597								
Open defecation	54/177	30.5	Ref			Ref		
Limited, Basic & Improved sanitation	63/420	15.0	0.4	0.3-0.6	<0.001	0.6	0.3-1.1	0.07
Hygiene assessment levels, n=597								
No facilities	21/69	30.4	Ref			Ref		
Limited	41/150	27.3	0.9	0.5-1.6	0.64	1.0	0.5-2.1	0.92
Basic	55/378	14.6	0.4	0.2-0.7	0.002	0.5	0.3-1.1	0.09

Table S6 Bivariate and multivariable analysis of associations between seroprevalence of anti-HEV, socio-demographics and WASH levels in participants aged less than 18 years old.

		Anti-HEV seroprevalence							
		Bivariate analysis					Multivariable analysis		
Variable		n/n total	% positive	OR	95% CI	p-value	OR	95% CI	p-value
District, n=617									
	Nakaiy	13/75	17.3	Ref			Ref		
	Mahaxay	48/246	19.5	1.2	0.6-2.3	0.67	1.2	0.6-2.5	0.58
	Bualapha	62/296	20.9	1.3	0.7-2.4	0.49	0.9	0.4-2.0	0.83
Sex, n=617									
	Male	52/289	18.0	Ref			Ref		
	Female	71/328	21.6	1.3	0.8-1.9	0.26	1.3	0.9-2.0	0.18
Age group, n=617									
	5-10	30/229	13.1	Ref			Ref		
	11-17	93/388	24.0	2.1	1.3-3.3	0.001	2.0	1.3-3.3	0.003
Ethnicity, n=617									
	Lao-Tai	53/304	17.4	Ref			Ref		
	Non-Lao-Tai	70/313	22.4	1.4	0.9-2.0	0.13	1.1	0.7-1.8	0.69
Wealth index measurement, n=597									
	Poor tertile	48/228	21.1	Ref			Ref		
	Middle tertile	37/178	20.8	1.0	0.6-1.6	0.95	1.2	0.7-2.0	0.60
	High tertile	34/191	17.8	0.8	0.5-1.3	0.40	1.0	0.5-1.8	0.89
Water assessment levels, n=597									
	Surface water	27/90	30.0	Ref			Ref		
	Unimproved water	4/21	19.0	0.5	0.2-1.8	0.32	0.4	0.1-1.5	0.22
	Limited, Basic & Improved water	88/486	18.1	0.5	0.3-0.9	0.01	0.5	0.3-0.9	0.03
Sanitation assessment levels, n=597									
	Open defecation	41/177	23.2	Ref			Ref		
	Limited, Basic & Improved sanitation	78/420	18.6	0.8	0.5-1.2	0.20	0.8	0.5-1.5	0.55
Hygiene assessment levels, n=597									
	No facilities	16/69	23.2	Ref			Ref		
	Limited	29/150	19.3	0.8	0.4-1.6	0.51	0.8	0.4-1.6	0.50
	Basic	74/378	19.6	0.8	0.4-1.5	0.49	0.9	0.4-1.8	0.71
